# Miscellaneous

**Published:** 1892-08

**Authors:** 


### MISCELLANEOUS.

### SIMPLE HOME REMEDIES.

Onion juice is one of the most effective remedies for earache. To prepare : Wrap a large onion in heavy wrapping paper, wet it thoroughly and roast in the coals. When tender, strip off the skin and squeeze out the juice by twisting in a thin cloth. Bottle and save for use. When needed, pour one or two drops in a spoon, warm a little and drop into the ear. Afterwards put in a bit of warm cotton to exclude the air. It rarely, if ever, fails to effect a speedy cure.

Lard and camphor are excellent remedies for a cold in the head or tightness of the chest, causing hard breathing. Soften a little fresh lard near the fire and stir into it an equal amount of camphor. Pour into a tin salve box or open mouthed bottle and cool as rapidly as possible, beating it all the time, that the camphor may be thoroughly incorporated with the lard, else the latter will settle to the bottom and the camphor remain on top.

If one's head be stopped up, rub all about nose, as well as forehead and temples. If a little be rubbed or snuffed up the nostrils it will be all the better. For tightness in chest rub thoroughly on throat and chest, and only a few applications will be needed to effect a cure.

For a bad cough, boil a tablespoonful of flaxseed for a few minutes in a cup of water. Strain, add the juice of a lemon, sweeten to taste and drink. The lemon cuts the phlegm, thus loosening the cough, while the flaxseed allays the inflammation.

---

### THE FECUNDITY OF FLIES.

| | |
|---|---|
| A common house fly lays four times during the summer season, each time an average of eighty eggs, which makes | 320 |
| One-half of these are supposed to be females, so that each of these four broods produces forty— | |
| 1.—First eighth, or forty females of the first brood, also lay four times in the course of the summer, which makes | 12,800 |
| The first eighth of these last, or 1600 females lay three times, making a "setting" of | 384,000 |
| The second eighth lay twice, or eggs to the number of | 256,000 |
| The third and fourth eighth lay at least once each, or | 256,000 |
| 2.—The second eighth, or the forty females of the second brood, lay three times and produce eggs to the amount of | 9,600 |
| One-sixth of these, or 1600 females, lay three times or eggs to the number of | 384,000 |
| The second sixth lay twice the eggs numbering | 256,000 |
| The third lay once, or | 128,000 |
| 3.—The third eighth or the forty females of the third brood, lay twice, or eggs to the number of | 6,400 |
| One-fourth of these, or 1600 females, lay twice more, which is | 256,000 |
| 4.—The fourth eighth or forty females of the fourth brood, have one laying period each, which produces eggs to the number of | 32,000 |
| Half of the these last, or 1600 females will also lay 80 eggs each, which gives us | 128,000 |
| Total product of a single pair of flies in one season | 2,080,320 |

It has been estimated that if each fly hatched should live to be four years of age, at the end of that time they would form a solid mass around the earth, extending to a height of fifty miles, or about the estimated thickness of our atmosphere.

---

### HOW VACCINE VIRUS IS OBTAINED.

The preparation used to produce what we call "vaccination," is known among medical men as vaccine virus, to produce which it is necessary to go through a surgical operation, the subject being a young cow or even a calf. After scarifying the belly of the animal (the parts having previously been shaved) the wound is inoculated with virus from an animal already in use. A sore is thus formed without lasting injury to the beast, and after a week or ten days a thin vaccid matter begins to flow from the abrasion. This pus or matter is the vaccine virus of commerce. Goose quills which have been scraped with a knife until they present a rough exterior are rubbed in this virus. The virus from one abrasion is sufficient to coat 10,000 quills, which after being so prepared are technically called "points." These points when ready for shipment look like ordinary goose quills, the virus coating not being visible to the naked eye.

---

KING ALFONSO XII. STILL UNBURIED.—I wonder, says a writer in the New York *Recorder*, how many people there are who are awarê of the strange fact that the late King Alfonso, of Spain, who died six years ago, is still unburied and awaiting his final interment in the tomb which has been prepared for his corpse, clothed only in a thin linen garment. The dead King lies on a slab of rock near a running spring of water in a cavern in the side of the mountain on the slope of which the grand old Escurial is built. There he will remain until his body has attained all the peculiar properties of a mummy, and then only will the ghastly object be placed in its niche in that marvelous jasper vault under the great dome of the Escurial Church, where only the remains of Spanish kings and of the mothers of kings are allowed to lie. Some bodies, notably that of Queen Isabella's profligate father, remained on the rock table for 20 and 25 years before they were in fit condition to be transferred to the vault. The name of this weird cavern is the "Pudrido," a name which is also misapplied to the vaults containing the bodies of the Infants and the Infantas.

REMOVING STAINS.—To remove fresh fruit stains from table linen, cover quickly with powdered starch, or pour boiling water from the tea-kettle upon them. Finely sifted wood ashes will remove medicine stains from silver spoons. Egg stains on silver can be taken off with fine salt and a damp cloth. On fabrics that will not be injured by it, soft soap will take out paint stains much better than benzine, chloroform, and similar cleaners. Hot cider-vinegar will remove paint stains from window glass, or nearly full strength oxalic acid used with a swab will produce the same effect. In using the latter, care must be taken that it does not touch the hands or the paint on the surrounding woodwork. If marble is discolored by grease or any other kind of stain, mix two parts of common washing soda with one part chalk, and one of ground pumice-stone. Make sure that all are finely powdered, and then make into a paste with water. Rub it well over the marble, and an hour or two later wash it off with plain soap and water. Another way of cleaning marble is to mix whiting and curd soap to a paste. Spread and leave for a couple of days, then wash it off, and the marble will be as white as possible.

A WONDERFUL STATEMENT.—No report concerning electricity, or the achievements of those working in the field which is covered thereby, is calculated to astonish the general reader. So much is claimed for it that it seems capable of accomplishing any thing. Among the recent discoveries which are authoritatively announced is the application of high voltage currents in the extirpation of cancer. A current of 500 mille amperes is sent through the growth, and by a few applications the malignant nature of the cancer is completely destroyed, and the tumor shrinks into a small fibrous mass which is absolutely inert. Dr. A. Wilbur Jackson has successfully used the current in several cases already reported at length, and Inglis Parsons, of London, also writes to the *British Medical Journal*, of his success in this operation. Dr. Jackson has become widely known as an expert electro-therapeutist, and has charge of that department in the Polypathic Medical Institute. He is also an authority in nervous and mental diseases.

NATURE'S FOOD FOR INFANTS.—It is not generally known that the foundation of many fatal diseases is laid during the first twelve months of an infant's life, chiefly by the substitution, in whole or in part, of artificial for natural food. Rickets, diseases of the mesenteric glands, water in the brain, and many other diseases so frequent in early life, are produced by this error. No mother possessing a tolerable share of health, and a good breast of milk, should feed her infant upon any thing else. The destructive practice of giving tops and bottoms, burnt flour, and the like, cannot be too much deprecated ; they are indigestible, and that which cannot be digested is poison to the delicate stomach and frame of an infant. Another source of injury is often experienced by a child at the breast, from the practice of many mothers, who, with a view of supporting their strength, during lactation, habituate themselves to the use of spirits, wine, or a double allowance of beer. This inflames the blood, and, of course, has a direct deleterious effect on the milk.

LITTLE GIRL BROWN.—The wonderful sweetness of little Boy Blue is a tale that by this time to no one is new, but his sweetness and light can't compare in renown with the saccharine graces of little Girl Brown. She is fair as an angel and sweet seventeen, is old Brown's only child and his heiress I ween, and her father's the richest old beggar in town, and she dotes on yours truly, does little Girl Brown.

A REMEDY FOR THE AGUE.—The white of an egg beat up in a pint of white-wine vinegar, taken when the trembling is expected to come on, operates strongly upon the patient, and has seldom or never failed to make a complete cure.

REMEDY FOR INDIGESTION.—Infusion of columba 6 oz., carbonate of potash 1 drachm, compound tincture of gentian 3 drachms. Mix. Dose : three tablespoonfuls each day an hour before dinner. The juice of a lemon taken immediately after the principal meal of the day is also beneficial.

BEDS, when occupied, should not be placed with one side close to the wall. In this position the sleeper's breath is thrown back and inhaled again, a most pernicious practice. Another objection is possible dampness from the wall. Let there be free circulation of air around the bed, especially if there are two occupants.

